# 1-(2,4,6-Trioxo-1,3-diazinan-5-yl­idene)thio­semicarbazide

**DOI:** 10.1107/S1600536812012007

**Published:** 2012-03-24

**Authors:** Viviane C. D. Bittencourt, Vanessa Carratu Gervini, Leandro Bresolin, Aline Locatelli, Adriano Bof de Oliveira

**Affiliations:** a Escola de Química e Alimentos, Universidade Federal do Rio Grande, Av. Itália km 08, Campus Carreiros, 96203-900, Rio Grande, RS, Brazil; b Departamento de Química, Universidade Federal de Santa Maria, Av. Roraima, Campus, 97105-900, Santa Maria, RS, Brazil; c Departamento de Química, Universidade Federal de Sergipe, Av. Marechal Rondon s/n, Campus, 49100-000, São Cristóvão, SE, Brazil

## Abstract

The title mol­ecule, C_5_H_5_N_5_O_3_S, is approximately planar, with a maximum deviation from the mean plane through the non-H atoms of 0.182 (3) Å for the amine N atom. In the crystal, mol­ecules are connected *via* N—H⋯O and N—H⋯S inter­actions, building a three-dimensional hydrogen-bonded network. Additionally, a weak intra­molecular N—H⋯O hydrogen bond is observed.

## Related literature

For the synthesis of alloxan-5-thio­semicarbazone, see: Beyer *et al.* (1956 ▶). For the anti­bacterial activity of alloxan-5-thio­semicarbazone against *Staphylococcus aureus* and *Escherichia coli*, see: Douros *et al.* (1973 ▶).
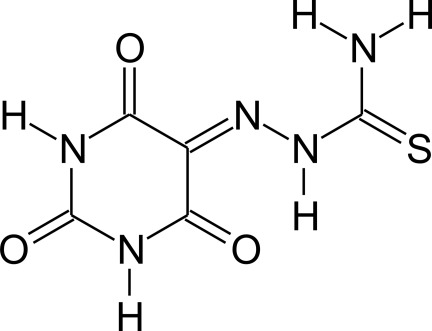


## Experimental

### 

#### Crystal data

C_5_H_5_N_5_O_3_S
*M* *_r_* = 215.20
Monoclinic, 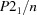

*a* = 10.6415 (8) Å
*b* = 7.3370 (6) Å
*c* = 11.160 (1) Å
β = 107.380 (5)°
*V* = 831.55 (12) Å^3^
*Z* = 4
Mo *K*α radiation
μ = 0.38 mm^−1^
*T* = 293 K
0.14 × 0.10 × 0.09 mm

#### Data collection

Bruker APEXII CCD diffractometer
Absorption correction: multi-scan (*SADABS*; Bruker, 2005 ▶) *T*_min_ = 0.949, *T*_max_ = 0.967
15454 measured reflections
1929 independent reflections
955 reflections with *I* > 2σ(*I*)
*R*_int_ = 0.090

#### Refinement

*R*[*F* ^2^ > 2σ(*F* ^2^)] = 0.056
*wR*(*F* ^2^) = 0.148
*S* = 1.00
1929 reflections
147 parameters
All H-atom parameters refined
Δρ_max_ = 0.27 e Å^−3^
Δρ_min_ = −0.39 e Å^−3^

### 

Data collection: *COSMO* (Bruker, 2005 ▶); cell refinement: *SAINT* (Bruker, 2005 ▶); data reduction: *SAINT* (Bruker, 2005 ▶); program(s) used to solve structure: *SHELXS97* (Sheldrick, 2008 ▶); program(s) used to refine structure: *SHELXL97* (Sheldrick, 2008 ▶); molecular graphics: *DIAMOND* (Brandenburg, 2006 ▶); software used to prepare material for publication: *publCIF* (Westrip, 2010 ▶).

## Supplementary Material

Crystal structure: contains datablock(s) I, global. DOI: 10.1107/S1600536812012007/nc2271sup1.cif

Structure factors: contains datablock(s) I. DOI: 10.1107/S1600536812012007/nc2271Isup2.hkl

Supplementary material file. DOI: 10.1107/S1600536812012007/nc2271Isup3.cml

Additional supplementary materials:  crystallographic information; 3D view; checkCIF report

## Figures and Tables

**Table 1:** Hydrogen-bond geometry (Å, °)

*D*—H⋯*A*	*D*—H	H⋯*A*	*D*⋯*A*	*D*—H⋯*A*
N5—H4⋯O2^i^	0.91 (4)	2.26 (4)	3.036 (4)	143 (3)
N2—H2⋯O3^ii^	0.86 (4)	1.98 (4)	2.837 (4)	173 (4)
N5—H5⋯O1^iii^	0.89 (5)	2.08 (5)	2.916 (4)	158 (4)
N4—H3⋯O1	0.88 (4)	2.01 (4)	2.631 (4)	126 (4)
N1—H1⋯O3^iv^	0.70 (4)	2.46 (4)	2.923 (4)	125 (4)
N1—H1⋯S1^v^	0.70 (4)	3.03 (4)	3.468 (4)	123 (4)

Symmetry codes: (i) 
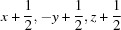
; (ii) 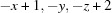; (iii) 
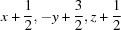
; (iv) 
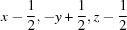
; (v) 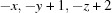.

**Fig. 1.**
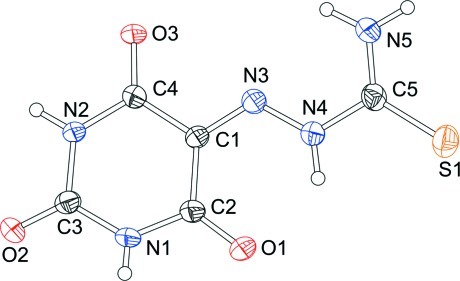
: The molecular structure of the title compound with labeling and displacement ellipsoids drawn at the 40% probability level.

**Fig. 2.**
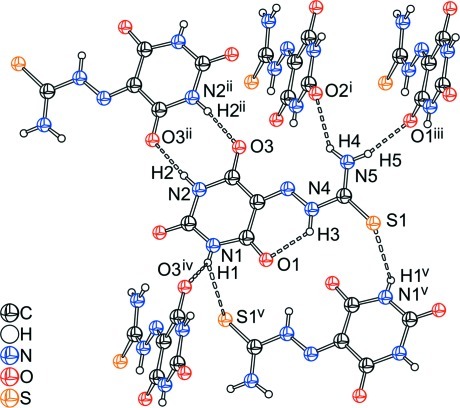
: The crystal structure of the title compound showing the molecules connected through N—H···S hydrogen bonds. Hydrogen bonding is indicated as dashed lines. Symmetry codes: (i) x + 1/2, -y + 1/2, z + 1/2; (ii) -x + 1/2, -y, -z + 2; (iii) x + 1/2, -y + 3/2, z + 1/2; (iv) x - 1/2, -y + 1/2, z - 1/2; (v) -x, -y + 1, -z + 2.

## supplementary crystallographic information

### Comment

Thiosemicarbazone derivatives have a wide range of biological properties. For example, an alloxan-thiosemicarbazone derivative shows antibacterial activity against several pathologic agents like *Staphylococcus aureus* and *Escherichia coli* (Douros *et al.*, 1973). As part of our study of thiosemicarbazone derivatives, we report herein the crystal structure of alloxan-5-thiosemicarbazone. In the title compound (Fig. 1), the molecule is planar and the maximal deviation from the least squares plane through all non-hydrogen atoms is observed for N5 (-0,1822 (30) Å). The mean deviations from the least squares planes for the alloxan fragment C1/C2/C3/C4/N1/N2/O1/O2/O3 and for the thiosemicarbazone fragment C5/N3/N4/N5/S1 amount to 0.0319 (23) Å for O3 and -0.0278 (26) Å for N4, respectively, and the dihedral angle between the two planes is 8,16 (17)°. The bond angles suggest *sp*^2^ hybridization for the C and N atoms and explain the planarity of the molecule. The crystal packing is stabilized by intermolecular N—H···O and N—H···S as well as intramolecular N—H···O hydrogen bonding building a three-dimensional H-bonded network (Fig. 2 and Table 1).

### Experimental

Starting materials were commercially available and were used without further purification. The synthesis was adapted from a procedure reported previously (Beyer *et al.*, 1956). The hydrochloric acid catalyzed reaction of alloxan monohydrate (6,25 mmol) and thiosemicarbazide (6,25 mmol) in ethanol (60 ml) was refluxed for 7 h. After cooling and filtering, crystals suitable for X-ray diffraction were obtained from a recrystallization in methanol.

### Refinement

All hydrogen atoms were localized in a difference density Fourier map. Their positions and isotropic displacement parameters were refined.

### Crystal data

**Table:**

C_5_H_5_N_5_O_3_S	*F*(000) = 440
*M**_r_* = 215.20	*D*_x_ = 1.719 Mg m^−^^3^
Monoclinic, *P*2_1_/*n*	Mo *K*α radiation, λ = 0.71073 Å
Hall symbol: -P 2yn	Cell parameters from 1283 reflections
*a* = 10.6415 (8) Å	θ = 2.3–19.9°
*b* = 7.3370 (6) Å	µ = 0.38 mm^−^^1^
*c* = 11.160 (1) Å	*T* = 293 K
β = 107.380 (5)°	Block, red
*V* = 831.55 (12) Å^3^	0.14 × 0.10 × 0.09 mm
*Z* = 4	

### Data collection

**Table:**

Bruker APEXII CCD diffractometer	1929 independent reflections
Radiation source: fine-focus sealed tube	955 reflections with *I* > 2σ(*I*)
Graphite monochromator	*R*_int_ = 0.090
φ and ω scans	θ_max_ = 27.6°, θ_min_ = 2.3°
Absorption correction: multi-scan (*SADABS*; Bruker, 2005)	*h* = −13→13
*T*_min_ = 0.949, *T*_max_ = 0.967	*k* = −9→9
15454 measured reflections	*l* = −14→14

### Refinement

**Table:**

Refinement on *F*^2^	Primary atom site location: structure-invariant direct methods
Least-squares matrix: full	Secondary atom site location: difference Fourier map
*R*[*F*^2^ > 2σ(*F*^2^)] = 0.056	Hydrogen site location: difference Fourier map
*wR*(*F*^2^) = 0.148	All H-atom parameters refined
*S* = 1.00	*w* = 1/[σ^2^(*F*_o_^2^) + (0.0543*P*)^2^ + 0.6101*P*] where *P* = (*F*_o_^2^ + 2*F*_c_^2^)/3
1929 reflections	(Δ/σ)_max_ < 0.001
147 parameters	Δρ_max_ = 0.27 e Å^−^^3^
0 restraints	Δρ_min_ = −0.39 e Å^−^^3^

### Special details

**Table:**

Geometry. All e.s.d.'s (except the e.s.d. in the dihedral angle between two l.s. planes) are estimated using the full covariance matrix. The cell e.s.d.'s are taken into account individually in the estimation of e.s.d.'s in distances, angles and torsion angles; correlations between e.s.d.'s in cell parameters are only used when they are defined by crystal symmetry. An approximate (isotropic) treatment of cell e.s.d.'s is used for estimating e.s.d.'s involving l.s. planes.
Refinement. Refinement of *F*^2^ against ALL reflections. The weighted *R*-factor *wR* and goodness of fit *S* are based on *F*^2^, conventional *R*-factors *R* are based on *F*, with *F* set to zero for negative *F*^2^. The threshold expression of *F*^2^ > σ(*F*^2^) is used only for calculating *R*-factors(gt) *etc*. and is not relevant to the choice of reflections for refinement. *R*-factors based on *F*^2^ are statistically about twice as large as those based on *F*, and *R*- factors based on ALL data will be even larger.

### Fractional atomic coordinates and isotropic or equivalent isotropic displacement parameters (Å^2^)

**Table:**

	*x*	*y*	*z*	*U*_iso_*/*U*_eq_	
S1	0.17866 (11)	0.94933 (14)	1.13122 (11)	0.0487 (4)	
O3	0.4802 (2)	0.2077 (3)	1.0689 (2)	0.0426 (8)	
O2	0.1726 (2)	−0.0867 (4)	0.7631 (3)	0.0486 (8)	
O1	0.0566 (2)	0.4570 (3)	0.8863 (3)	0.0456 (8)	
N5	0.4116 (3)	0.7883 (5)	1.1940 (3)	0.0412 (9)	
N3	0.3183 (3)	0.4951 (4)	1.0600 (3)	0.0335 (8)	
N4	0.2392 (3)	0.6328 (4)	1.0593 (3)	0.0385 (9)	
N2	0.3282 (3)	0.0686 (4)	0.9108 (3)	0.0321 (8)	
N1	0.1178 (3)	0.1858 (4)	0.8272 (3)	0.0356 (9)	
C5	0.2851 (4)	0.7858 (5)	1.1334 (4)	0.0340 (9)	
C4	0.3687 (3)	0.2088 (5)	0.9943 (4)	0.0322 (9)	
C3	0.2034 (3)	0.0473 (5)	0.8282 (4)	0.0329 (9)	
C1	0.2734 (3)	0.3545 (4)	0.9874 (3)	0.0294 (9)	
C2	0.1418 (3)	0.3399 (5)	0.8981 (3)	0.0335 (9)	
H4	0.465 (4)	0.694 (5)	1.189 (3)	0.043 (12)*	
H2	0.382 (4)	−0.019 (5)	0.912 (3)	0.040 (12)*	
H5	0.440 (5)	0.887 (7)	1.240 (5)	0.083 (17)*	
H3	0.156 (5)	0.639 (6)	1.015 (4)	0.072 (15)*	
H1	0.054 (4)	0.175 (5)	0.785 (4)	0.044 (14)*	

### Atomic displacement parameters (Å^2^)

**Table:**

	*U*^11^	*U*^22^	*U*^33^	*U*^12^	*U*^13^	*U*^23^
S1	0.0504 (7)	0.0356 (6)	0.0614 (8)	0.0045 (5)	0.0185 (5)	−0.0041 (6)
O3	0.0296 (14)	0.0404 (15)	0.0424 (17)	0.0099 (12)	−0.0126 (13)	−0.0101 (13)
O2	0.0382 (15)	0.0385 (17)	0.057 (2)	0.0004 (12)	−0.0034 (14)	−0.0207 (14)
O1	0.0344 (15)	0.0366 (16)	0.0530 (19)	0.0122 (13)	−0.0066 (13)	−0.0042 (14)
N5	0.034 (2)	0.031 (2)	0.052 (2)	−0.0026 (16)	0.0027 (17)	−0.0090 (18)
N3	0.0344 (18)	0.0289 (17)	0.0325 (19)	0.0029 (14)	0.0027 (14)	−0.0003 (14)
N4	0.0332 (19)	0.0305 (18)	0.044 (2)	0.0037 (15)	0.0001 (17)	−0.0064 (16)
N2	0.0257 (17)	0.0281 (17)	0.034 (2)	0.0058 (14)	−0.0034 (14)	−0.0037 (14)
N1	0.0248 (18)	0.0354 (19)	0.037 (2)	0.0010 (15)	−0.0055 (16)	−0.0062 (16)
C5	0.039 (2)	0.0248 (19)	0.037 (2)	−0.0024 (17)	0.0101 (18)	0.0021 (17)
C4	0.0299 (19)	0.030 (2)	0.033 (2)	0.0011 (16)	0.0035 (17)	−0.0035 (17)
C3	0.027 (2)	0.033 (2)	0.036 (2)	0.0024 (17)	0.0047 (17)	0.0017 (19)
C1	0.0280 (19)	0.0268 (19)	0.029 (2)	0.0048 (15)	0.0015 (16)	0.0018 (16)
C2	0.029 (2)	0.032 (2)	0.033 (2)	0.0023 (17)	0.0000 (17)	−0.0010 (17)

### Geometric parameters (Å, º)

**Table:**

S1—C5	1.645 (4)	N4—H3	0.88 (4)
O3—C4	1.229 (4)	N2—C4	1.368 (4)
O2—C3	1.208 (4)	N2—C3	1.380 (4)
O1—C2	1.227 (4)	N2—H2	0.86 (4)
N5—C5	1.314 (4)	N1—C2	1.360 (5)
N5—H4	0.91 (4)	N1—C3	1.362 (5)
N5—H5	0.89 (5)	N1—H1	0.70 (4)
N3—C1	1.311 (4)	C4—C1	1.460 (5)
N3—N4	1.313 (4)	C1—C2	1.460 (5)
N4—C5	1.394 (4)		
			
C5—N5—H4	121 (2)	N5—C5—S1	126.3 (3)
C5—N5—H5	115 (3)	N4—C5—S1	117.5 (3)
H4—N5—H5	123 (4)	O3—C4—N2	120.1 (3)
C1—N3—N4	119.1 (3)	O3—C4—C1	123.7 (3)
N3—N4—C5	120.4 (3)	N2—C4—C1	116.2 (3)
N3—N4—H3	126 (3)	O2—C3—N1	122.8 (3)
C5—N4—H3	114 (3)	O2—C3—N2	121.8 (3)
C4—N2—C3	125.8 (3)	N1—C3—N2	115.4 (3)
C4—N2—H2	119 (2)	N3—C1—C2	125.4 (3)
C3—N2—H2	115 (2)	N3—C1—C4	115.1 (3)
C2—N1—C3	127.3 (3)	C2—C1—C4	119.5 (3)
C2—N1—H1	117 (3)	O1—C2—N1	121.0 (3)
C3—N1—H1	116 (3)	O1—C2—C1	123.3 (3)
N5—C5—N4	116.1 (3)	N1—C2—C1	115.7 (3)
			
C1—N3—N4—C5	−178.6 (4)	O3—C4—C1—N3	5.6 (6)
N3—N4—C5—N5	5.3 (5)	N2—C4—C1—N3	−174.0 (3)
N3—N4—C5—S1	−177.8 (3)	O3—C4—C1—C2	−178.0 (4)
C3—N2—C4—O3	175.4 (4)	N2—C4—C1—C2	2.5 (5)
C3—N2—C4—C1	−5.1 (6)	C3—N1—C2—O1	178.4 (4)
C2—N1—C3—O2	178.7 (4)	C3—N1—C2—C1	−1.7 (6)
C2—N1—C3—N2	−0.4 (6)	N3—C1—C2—O1	−3.5 (6)
C4—N2—C3—O2	−175.0 (4)	C4—C1—C2—O1	−179.6 (4)
C4—N2—C3—N1	4.1 (6)	N3—C1—C2—N1	176.6 (4)
N4—N3—C1—C2	3.5 (6)	C4—C1—C2—N1	0.6 (5)
N4—N3—C1—C4	179.7 (3)		

### Hydrogen-bond geometry (Å, º)

**Table:**

*D*—H···*A*	*D*—H	H···*A*	*D*···*A*	*D*—H···*A*
N5—H4···O2^i^	0.91 (4)	2.26 (4)	3.036 (4)	143 (3)
N2—H2···O3^ii^	0.86 (4)	1.98 (4)	2.837 (4)	173 (4)
N5—H5···O1^iii^	0.89 (5)	2.08 (5)	2.916 (4)	158 (4)
N4—H3···O1	0.88 (4)	2.01 (4)	2.631 (4)	126 (4)
N1—H1···O3^iv^	0.70 (4)	2.46 (4)	2.923 (4)	125 (4)
N1—H1···S1^v^	0.70 (4)	3.03 (4)	3.468 (4)	123 (4)

Symmetry codes: (i) *x*+1/2, −*y*+1/2, *z*+1/2; (ii) −*x*+1, −*y*, −*z*+2; (iii) *x*+1/2, −*y*+3/2, *z*+1/2; (iv) *x*−1/2, −*y*+1/2, *z*−1/2; (v) −*x*, −*y*+1, −*z*+2.
